# A population-based screening study for cardiovascular diseases and diabetes in Danish postmenopausal women: acceptability and prevalence

**DOI:** 10.1186/s12872-018-0758-8

**Published:** 2018-02-05

**Authors:** Marie Dahl, Lars Frost, Rikke Søgaard, Ib Christian Klausen, Vibeke Lorentzen, Jes Lindholt

**Affiliations:** 1 Cardiovascular Research Centre, Regional Hospital Central Denmark, Heibergs Allé 4, 8800 Viborg, Denmark; 2 Department of Clinical Medicine, Aarhus University, 8000 Aarhus, Denmark; 3 Department of Public Health and Department of Clinical Medicine, Aarhus University, 8000 Aarhus, Denmark; 4 Centre for Research in Clinical Nursing, Viborg, Denmark; 5 Department of Cardiothoracic and Vascular Surgery, Odense University Hospital, 5000 Odense, Denmark

**Keywords:** Observational study, Screening, Peripheral arterial diseases, Abdominal aortic aneurysm, Diabetes mellitus type 2, Hypertension, Atrial fibrillation, Carotid plaque

## Abstract

**Background:**

Reducing women’s cardiovascular risk and the economic costs associated with cardiovascular diseases (CVD) and diabetes (DM) continues to be a challenge. Whether a multifaceted CVD screening programme is beneficial as a preventive strategy in women remains uncertain. The aim of this study was to investigate the prevalence of CVD and DM as well as the acceptability toward screening and preventive actions.

**Methods:**

An observational study was performed among all women born in 1936, 1941, 1946 and 1951 living in Viborg Municipality, Denmark, from October 2011. In total, 1984 were invited to screening for abdominal aortic aneurysm (AAA), peripheral arterial disease (PAD), carotid plaque (CP), hypertension (HT), atrial fibrillation (AF), DM and dyslipidaemia. Participants with positive tests were offered prophylactic intervention including follow-up consultations in case of AAA, PAD and/or CP. Participants with AAA ≥ 50 mm were referred to specialists in vascular surgery. Women with AF or potential familial hypercholesterolaemia (FH) were referred to cardiology work-up.

**Results:**

Among those invited, 1474 (74.3%) attended screening, but the attendees’ share decreased with increasing age groups (*p* < 0.001). AAA was diagnosed in 10 (0.7%) women, PAD in 101 (6.9%) and CP in 602 (40.8%). The percentage of women with these conditions rose with increasing age group (*p* < 0.05). Unconfirmed potential HT was observed in 94 (6.4%), unknown AF in 6 (0.4%), DM in 14 (1%) and potential FH in 35 (2.4%). None of these findings differed across age groups. Among the 631 women diagnosed with AAA, PAD and/or CP, 182 (28.8%) were already in antiplatelet and 223 (35.3%) in lipid-lowering therapy prior to screening. Antiplatelet therapy was initiated in 215 (34.1%) and lipid-lowering therapy in 191 (30.3%) women. Initiation of antiplatelet and lipid-lowering therapy was further recommended to 134 (21.2%) and 141 (22.4%) women, respectively, who hesitated to follow the recommendation.

**Conclusions:**

The study recorded an acceptable total attendance rate, even though a significantly lower attendance rate was observed in the eldest women. The identified hesitation towards prophylactic therapy may affect the rationale and effectiveness of CVD screening, and hesitation seems a critical issue that should be addressed in the design of future screening programmes.

## Background

Cardiovascular diseases (CVD) and diabetes (DM) are major causes of increased morbidity and mortality in the Western world and much effort has been invested in designing optimal and cost-effective prevention strategies. Such strategies include early screening of at-risk populations. For example, solitary screening for abdominal aortic aneurysm (AAA) in elderly men has been proven cost-effective [1, 2] and has been introduced in several countries for elderly men, but not for elderly women. A combined screening programme for AAA, peripheral arterial disease (PAD) and hypertension (HT) among men aged 65–74 years demonstrated high prevalences of these diseases and was beneficial for detection of prevalent cases [3]. However, the results of that study cannot be extrapolated to the general population since CVD and DM seem to affect men and women differently. These gender differences affect the natural history of the diseases, disease prevalences and the proportion of the population that may benefit from initiation of prophylactic actions. Evidence indicates a lower prevalence of AAA among women than among men, whereas AAA rupture rates are almost fourfold higher in women than in men [4], which may counter-balance the lower prevalence from a cost-effectiveness point of view [5]. In addition, women diagnosed with atrial fibrillation (AF) have a higher risk of stroke [6]; yet women are less likely than men to receive sufficient oral anticoagulant therapy (OAC) [7]. Furthermore, women with stroke have a poorer functional outcome than men [8]. Similarly, women with PAD tend to become more immobilised than men because they experience a more rapid functional decline [9]. Additionally, women with DM have a higher risk of CVD than men with DM [10, 11]. Gender-related differences may therefore hinder extrapolation of the benefits and effects of CVD screening among men to a female population**.** The aim of this screening study was to investigate the prevalence of CVD and DM in women as well as the acceptability toward screening and preventive actions.

## Methods

### Study population

This was an observational screening study among all women born in 1936, 1941, 1946 and 1951 living in the Municipal of Viborg, Denmark. The study population was identified in the Danish Civil Registration System from October 2011 to January 2013; a total of 1984 women were included in the study.

### The screening programme

The screening programme was offered within a hospital setting. The screening tests were performed by the first author, who had experience with CVD screening, including ultrasonography.

A secretary sent out the invitations, which included a plain language statement, a small questionnaire, a pre-booked time for screening and details allowing the invitees to reschedule or decline the invitation by phone, email or surface mail. The screening sessions were scheduled with three women per hour. Women who did not attend their screening programme booking and had not declined the invitation were re-invited once. Non-responders were invited on specific days with 10 women being booked per hour. Fridays were allocated to follow-up on positive screening findings, including initiation of prophylactic treatments and counselling.

Preformatted forms and questionnaires were used for data collection. The screening programme included examination for AAA, PAD, carotid plaque (CP), HT, AF, DM and hypercholesterolaemia/familial hypercholesterolaemia (FH). Information was also collected concerning self-reported medical history, lifestyle parameters, height, weight, use of pharmacological drugs, family predispositions, walking-related pain assessed by The Walking Impairment Questionnaire, and self-rated quality of life assessed by the EuroQol’s EQ-5D-3 L.

### The screening programme procedure, diagnosis criteria and confirmation of findings

- Two-dimensional B-mode ultrasonography of the abdominal aorta was performed in a standardised and validated manner [12]. An inner-to-inner measurrement was used during the peak of cardiac systole. AAA was defined as an anterior–posterior (AP) aortic diameter ≥ 30 mm. AAA 30–50 mm were referred for an annual ultrasound scan and AAA ≥ 50 mm for contrast computed tomography (CT) and vascular surgical consultation. Aorta ectasia was defined as an AP diameter between 25 and 29 mm.
- Ankle-brachial pressure index (ABPI) was measured using a validated method [13]. The arm with the highest systolic pressure was used. Arm blood pressures (BP) were measured synchronously with BP in the tibialis posterior and dorsalis pedis arteries. The mean of the pedal systolic BPs was divided by the brachial systolic pressure. This procedure was repeated on the other leg. PAD was defined as an ABPI < 0.9 or ≥1.4 and verified by a re-measurement within 7–14 days.
- Two-dimensional B-mode and colour Doppler ultrasound imaging was performed in longitudinal and cross-sectional mode of the common (CCA), internal and external carotid arteries. Plaque was defined as a focal structure that encroaches into the arterial lumen of ≥0.5 mm or ≥50% of the surrounding vessel wall. Furthermore, the carotid intima-media thickness (cIMT) was measured based on the best measure images of the CCA far wall for each side at a plaque-free area. However, a cIMT ≥1.5 mm was not classified as plaque. Women with plaque were invited for a re-scan to verify the plaque measurements.
- BP was measured synchronously in both arms. In total, 3 BP recordings were obtained in the arm with the highest systolic pressure. Women without known HT and BP ≥ 160/100 mmHg were recommended BP controls. Home measurement was the prefered method for clarfication.
- The heart rhythm was heard in the Doppler speaker during ABPI measurements. In case of an unknown abnormal heart rhytm, an electrocardiogram (ECG) was performed to identify potential AF. If the ECG verified AF, the strategy for further investigation and treatment was dicussed with one of the two cardiologists in the project team.
- Glycated haemoglobin (HbA1c) was analysed at the bedside, and DM was diagnosed if HbA1c was ≥48 mmol/mol. Women diagnosed with DM were recommended to contact their general practiotioner (GP) for further interventions.
- Total cholesterol (TC) was measured at the bedside. If TC was ≥7.5 mmol/L, all lipid parameters were measured and analysed at the laboratory. Women with TC ≥ 7.5 mmol/L and LDL ≥ 5.5 mmol/L were referred to further diagnostic clarification and treatment of potential FH by a cardiologist. In case of AAA, PAD and/or CP, statin therapy (40 mg/day simvastatin) was recommended if TC exceeded 4.0 mmol/L.

The screening result was communicated immediately to the participants in writing as well as verbally. Additionally, the secretary informed the GPs digitally. Women with AAA, PAD and/or CP were re-invited to confirm the findings and provide counselling, among others involving risk modification by encouraging the participant to start antiplatelet and lipid-lowering therapy (if these had not already been initiated or a suspicion of a relative contraindication existed), physical exercises, smoking cessation and diet. Further supportive counselling was offered by the preventive consultants in Viborg Municipality; the women were recommended to bring a relative to these counselling sessions to enhance motivation and accountability*.*

### Statistical analysis

In Tables 1 and 2, baseline charateristics and screening results are reported as frequencies (%) or medians with interquartile ranges. Tests for differences across age groups for categorical variables were analysed by the the chi-squared (*x*^2^) test. Differences of parametric data (TC and diastolic BP) were estimated by one-way ANOVA. Differences of non-parametric data (Body Mass Index, HbA1c and systolic BP) were analysed by Kruskal-Wallis test. In Tables 3 and 4, frequencies (%) are reported. *P* values in the tables refer to the used statistical test for difference across age groups. Any *p* value < 0.05 was considered statisctically significant. Data were analysed using the software programme STATA, version 13.1 (Statacorp, College Station, TX, USA).

**Table 1:** Characteristics of the study population

	Age group 1 (*n* = 592)	Age group 2 (*n* = 622)	Age group 3 (*n* = 433)	Age group 4 (*n* = 337)	*p* value
Age (years)	61 (0.8)	66 (0.8)	71 (0.9)	76 (0.8)	NA
Current smoker (n)	88 (18.3)	68 (14.4)	56 (17.1)	20 (10.5)	0.062
Self-reported comorbidity (n)					
Stroke	16 (3.3)	20 (4.2)	28 (8.5)	23 (12.1)	< 0.001
Myocardial infarction	11 (2.3)	13 (2.7)	5 (1.5)	9 (4.7)	0.194
Diabetes mellitus	32 (6.6)	45 (9.5)	27 (8.2)	12 (6.3)	0.278
Hypertension	139 (28.8)	196 (41.4)	160 (48.8)	109 (57.4)	< 0.001
Atrial fibrillation	10 (2.1)	14 (3.0)	13 (4.0)	10 (5.3)	0.151
Chronic obstructive pulmonary disease	20 (4.2)	29 (6.1)	25 (7.6)	16 (8.4)	0.136
Self-reported family predisposition (n)					
Stroke	141 (29.3)	126 (26.6)	92 (28.1)	49 (25.8)	0.406
Myocardial infarction	153 (31.7)	173 (36.5)	107 (32.6)	55 (29.0)	0.392
Diabetes mellitus	97 (20.1)	103 (21.7)	83 (25.3)	36 (19.0)	0.529
Hypertension	252 (52.3)	233 (49.2)	169 (51.5)	79 (41.6)	0.329
Peripheral arterial disease	96 (19.9)	80 (16.9)	63 (19.2)	38 (20.0)	0.688
Abdominal aortic aneurysm	16 (3.3)	24 (5.1)	12 (3.7)	5 (2.6)	0.570

Except for age (*n* = 1984), the characteristics are available only for those who attended screening (*n* = 1474). Values are medians (50th centile) with interquartile ranges or n (%). *P* value refers to the *x*^2^ test for difference across age groups. Family predisposition was defined as mother, father, sister or brother

**Table 2:** Screening results

	Age group 1 (*n* = 592)	Age group 2 (*n* = 622)	Age group 3 (*n* = 433)	Age group 4 (*n* = 337)	*p* value
Attandance (n)					
At pre-booked date	382 (64.5)	400 (64.3)	272 (62.8)	154 (45.7)	< 0.001
After re-scheduling	83 (14)	62 (10)	49 (11.3)	23 (6.8)	0.006
After new invitation	17 (3.5)	12 (2.5)	7 (2.1)	13 (6.8)	0.021
Total attendance	482 (81.4)	474 (76.2)	328 (75.8)	190 (56.4)	< 0.001
Rejected invitation	49 (8.3)	80 (12.9)	54 (12.5)	82 (24.3)	< 0.001
No response	61 (10.3)	68 (10.9)	51 (11.8)	65 (19.3)	< 0.001
Test results (median)					
Body Mass Index (kg/m^2^)	25.0 (6.4)	25.3 (6.1)	25.6 (5.5)	25.8 (5.4)	0.190
Total-cholesterol (mmol/L)	5.9 (1.3)	5.8 (1.6)	5.8 (1.5)	5.6 (1.4)	0.008
Glycated haemoglobin (mmol/mol)	34 (6)	34 (5)	35 (6)	36 (5)	< 0.001
Systolic blood pressure (mmHg)	141 (26)	145 (28)	147 (25)	147 (34)	< 0.001
Diastolic blood pressure (mmHg)	81 (13)	80 (13)	78 (13)	76 (14)	< 0.001
Disease prevalence (n)					
Abdominal aortic aneurysm	0	3 (0.6)	3 (0.9)	4 (2.1)	0.025
Aortic ectasies	1 (0.2)	3 (0.6)	7 (2.1)	1 (0.5)	0.022
Peripheral arterial disease	15 (3.1)	26 (5.5)	30 (9.2)	30 (15.8)	< 0.001
Carotid plaque	148 (30.7)	188 (39.7)	161 (49.1%)	105 (55.3)	< 0.001
Potential hypertension	32 (6.6)	29 (6.1)	22 (6.7)	11 (5.8)	0.964
Potential familial hypercholesterolemia	14 (2.9)	13 (2.7)	4 (1.2)	4 (2.1)	0.422
Atrial fibrillation	1 (0.2)	3 (0.6)	1 (0.3)	1 (0.5)	0.748
Diabetes mellitus	6 (1.2)	3 (0.6)	5 (1.5)	0	0.270

The test results are available only for those who attended screening (*n* = 1474). Values are n (%) or medians (50th centile) with interquartile ranges. For categorical variables *p* value refers to the *x*^2^ test for difference across age groups. For continuous variables the *p* value refers to one-way ANOVA or Kruskal-Wallis test

**Table 3:** Status on statin therapy for women diagnosed with AAA, PAD and/or CP

	Age group 1 (*n* = 153)	Age group 2 (*n* = 197)	Age group 3 (*n* = 170)	Age group 4 (*n* = 111)	*p* value
Already in therapy	52 (34)	65 (33)	60 (35.3)	46 (41.4)	0.493
Referred to cardiologist	6 (3.9)	7 (3.6)	4 (2.4)	1 (0.9)	0.447
Potential contraindication	1 (0.7)	1 (0.5)	2 (1.2)	0	0.670
Total cholesterol < 4.0 mmol/L	5 (3.3)	10 (5.1)	4 (2.4)	4 (3.6)	0.568
Initiation of therapy	42 (27.5)	57 (28.9)	61 (35.9)	31 (27.9)	0.312
Hesitation to accept therapy	37 (24.2)	45 (22.8)	36 (21.2)	23 (20.7)	0.890
No response	10 (6.5)	12 (6.1)	3 (1.8)	6 (5.4)	0.164

Values are n (%). *P* value refers to the *x*^2^ test for difference across age groups

**Table 4:** Status on antiplatelet therapy for women diagnosed with AAA, PAD and/or CP

	Age group 1 (*n* = 153)	Age group 2 (*n* = 197)	Age group 3 (*n* = 170)	Age group 4 (*n* = 111)	*p* value
Already in therapy	36 (23.5)	48 (24.4)	54 (31.8)	44 (39.6)	0.012
Referred to cardiologist	2 (1.3)	0	1 (0.6)	0	0.289
Potential contraindication	15 (9.8)	15 (7.6)	12 (7.1)	16 (14.4)	0.157
Initiation of therapy	55 (36)	69 (35)	64 (37.7)	27 (24.3)	0.112
Hesitation to accept therapy	35 (22.9)	46 (23.4)	33 (19.4)	20 (18)	0.615
No response	10 (6.5)	19 (9.6)	6 (3.5)	4 (3.6)	0.059

Values are n (%). *P* value refers to the *x*^2^ test for difference across age groups

## Results

In all, 1984 women were invited for screening of whom 1474 (74.3%) attended. Table 1 shows the characteristics of the study population. The attendance rate decreased markedly with age (*p* < 0.001). Table 2 shows the attendance rates, the tests results and disease prevalences across age groups.

AAA, PAD and/or CP were found in 631 women. AAA was diagnosed in 10 women (0.7%; 95% CI: 0.33; 1.24); two AAAs were above 50 mm. The prevalence of AAA was significantly associated with age (*p* = 0.025). Aortic ecstasies were found in 12 women (0.8%; 95% CI: 0.42; 1.42). The mean aortic diameter for the total screened population was 16.5 mm (95% CI: 16.3; 16.6).

PAD was diagnosed in 101 women yielding a prevalence of 6.9% (95% CI: 5.62; 8.26); only one had an ABPI ≥1.4. In women with a median age of 61 years, the prevalence of PAD was 3.1%, increasing to 5.5% in age group 2, to 9.2% in age group 3 and to 15.8% in age group 4, indicating a significant association between PAD and age (*p* < 0.001). After adjusting for smoking habits, DM, known HT, previous stoke and myocardial infarction, the association between PAD and age remained significant when comparing age group 1 to age group 3 (p < 0.001) and 4 (p < 0.001). Significant association was also found between PAD and smoking habits (p < 0.001). Nearly half (43.6%) reported limited walking distance with the characteristics of intermittent claudication (95% CI: 33.7; 53.8).

CP was found in 602 women, yielding a prevalence of 40.8% (95%CI: 38.32; 43.40). The prevalence of plaque was significantly associated with age (*p* < 0.001); the prevalence increased from 30.7% in age group 1 to 55.3% in age group 4.

Some women were diagnosed with more than one condition. The presence of CP was identified in 73 of the women with PAD (72.3%; 95% CI: 62.48; 80.72). The odds ratio for PAD in women with plaque was 2.7 after adjusting for confounders (95% CI: 1.69; 4.37, *p* < 0.001). The mean ABPI in women with plaque was 0.99 versus 1.05 in women without plaque. However, in women with plaque, the mean ABPI decreased from 1.02 in age group 1 to 0.96 in age group 4. In women diagnosed with AAA, PAD or/and CP, 42 (6.7%) women were found to have potential HT.

Known HT was reported by 41% (95% CI: 38.45; 43.54) and the frequency increased from 28.8% in the youngest group to 57.4% in the oldest group of women (*p* < 0.001). Possible HT based on BP ≥ 160/100 mmHg was found in 94 women, corresponding to 6.4% of the screened population (95% CI: 5.18; 7.75); in this group, 44 (46.8%) had verified HT above 160/100 mmHg, 19 (20.2%) had mild HT, 24 (25.5%) had normalized BP and seven (7.4%) women missed follow-up.

Among 1414 of the attending women, 47 reported current or previous AF, corresponding to a prevalence of 3.3% [95% CI: 2.45; 4.40]; 19 (40.4%) were currently in OAC. Women without sufficient OAC were referred to cardiological counselling for assessment of OAC indication. Further, six women were diagnosed with AF, corresponding to 0.4% (95% CI: 0.15; 0.88).

Among 1470 of the screened women, 116 reported known DM (7.9%; 95% CI: 6.56; 9.38). HbA1c levels ≥48 mmol/mol were found in 14 women (1%; 95% CI: 0.52; 1.59).

Among the screened population, use of lipid-lowering therapy was reported by 29.6% and antiplatelets like low-dose aspirin (ASA) and clopidogrel were used by 18.3% and 2.6%, respectively. In total, 35 women (2.4%; 95% CI: 1.66; 3.29) were referred to cardiological consultation for clarification of potential FH. In women diagnosed with AAA, PAD or/and CP (*n* = 631), 35.3% received lipid-lowering therapy prior to screening. In 30.3%, statin therapy was initiated at the follow-up consultation and a further 22.4% were recommended statin, but expressed hesitation towards lipid-lowering therapy (see Table 3 for details). In women with AAA, PAD and/or CP, 24.3% reported receiving ASA, 5.1% clopidogrel and 3.5% OAC prior to screening. In total, 28.8% received one or a combination of antiplatelets. ASA was initiated in 34.1%, and further 21.2% expressed hesitation towards ASA (see Table 4 for details).

## Discussion

This study represents a multifaceted screening programme for CVD and DM in elderly women, and offers insights into the acceptability of screening and the prevalence of these diseases in a previously little investigated group. The study recorded an acceptable total attendance rate of 74.3%, even though a significantly lower attendance rate was observed in the eldest women. The main findings were as follows: prevalence of AAA (0.7%), PAD (6.9%), CP (40.8%), potential HT (6.4%), unknown AF (0.4%), unknown DM (1%) and potential FH (2.4%). Lipid-lowering therapy was initiated in 30.3% and antiplatelet therapy in 34.1% of the women diagnosed with AAA, PAD and/or CP; yielding a coverage percentage of 65.6 for lipid-lowering thearpy and 62.9 for antiplatelet therapy.

The present study illuminated multiple factors that may affect the rationale of a combined screening programme:

1. Response rate.
  Acceptance was significantly lower in the group with a median age of 76 years. It may therefore be beneficial to not include this age group in future screening programmes. As the prevalence of disease increases with age, it may therefore still make sense to extend the offer of screening to this group despite its lower attendance rate. Furthermore, non-attendance is related to lifestyle factors especially smoking [14]. Consequently, this age group is likely to be more prone to need prevention. The decrease in attendance rate among the oldest women seems not to be gender-specific. Hence, Lindholt et al. found similar trend among men [15]. Alongside the screening programme, we have interviewed 10 non-attendees to explore reasons for non-attendance. Finding the screening offer personally irrelevant was revealed as the main theme for non-attendance. This perception was, however, found to be changeable especially by newfound awareness of the screening programme and the potential benefits of attending [16].
2. Disease prevalence.
  - In accordance with previous studies, we found a lower prevalence of AAA in women than in men [17]. Even so, screening for AAA may still be cost-effective in women because they are at higher risk of aneurysm rupture than men [4]. A Markov decision-analytic model from 2006 indicated that the cost-effectiveness of screening for AAA in female was rather insensitive to variation when the prevalence exceeded 1% [5]. However, this calculation was made based on a solitary screening offer and may therefore not apply in the present study, which offered a multifaceted screening offer. The threshold for AAA is derived from men, where 30 mm represent an approximately 50% enlargement of the infrarenal aorta [18]. However, gender-specific mean maximum diameter of the aorta is considerable; in Danish elderly men without and with family history of AAA, the mean for the total screened population was 19.07 mm and 20.5 mm, respectively [19] compared with 16.5 mm and 17.2 mm in the present study. In case of decreasing the threshold for AAA in women to 25 mm, the prevalence in the present study would increase by 0.8%. Only long-term follow-up will determine whether a lower threshold will compensate for the higher rupture rate in women as well as the clinical relevance.
  - In Danish men who were screened for PAD, the prevalence was 8.0% in the 65-year-olds, increasing to 15.6% in 74-year-olds. A similar age-related increase in PAD was found in the present study: In women with median ages of 66 and 76 years, the prevalences of PAD were 5.5% and 15.8%, respectively.
  - According to a systematic review, pulse palpation has a high sensitivity, but a relatively low specificity for identifying undiagnosed AF [20]. In the present study, the prevalence of undiagnosed AF was only 0.4%, which questions the efficacy of assessment of the heart rhythm during ABPI measurement – particularly so because the prevalence of undiagnosed AF based on the traditional pulse palpation approach (and ECG in case of an irregular pulse) was 1.6% in a population aged 65 or older [21].
  - In the screening programme for AAA, PAD and HT among Danish men aged 65–74 years, the prevalence of potential HT at a cut-off value of 160/100 mmHg was 10.5% [3] versus 6.4% in the present study. In contrast hereto, no gender-specific difference was found in relation to self-reported, known HT in the two Danish screening programmes.
3. The rationale of screening for CP.
  CP was found in 40.8% of the women. Carotid duplex ultrasound adds information beyond traditional CVD risk stratification that may help to decide the potential need for primary prevention [22]. Evidence suggests that adding CP information improves the CHD risk prediction [23, 24] and statin therapy has been found to promote delipidation of CP [25].
4. Hesitation to comply with recommended preventive actions.
  Approximately 22% of the women who were recommended prophylactic therapy hesitated to comply with the recommendations with a trend towards a decrease in hesitation with older age. Medical records showed that the women preferred to reduce cholesterol levels through dietary changes rather than by using medicine or discussing ASA/statin use with their GPs. Unfortunately, no follow-up data on adherence to follow-up at GPs are available for the women who expressed this hesitation. The observed hesitation towards prophylactic ASA/statin use might reduce the potential health benefits of diagnosing pre-manifest and manifest diseases and, consequently, reduce the effectiveness of screening programmes. However, it is likely that methods aiming to increase acceptability could be developed, and a focus on this issue is indicated in the future. In men who were screened positive for PAD, hesitation towards prophylactic medicine has also been reported [3]. Whether the underlying reasons for hesitation are gender-specific calls for further study.
5. Chosen cut-off values.
  Selecting cut-off values at 140/90 mmHg for BP and 42 mmol/mol for HbA1c might lead to an unacceptably high number of false-positive findings with associated psychological and societal costs. At the mentioned cut-off values, potential HT would have been observed in 178 women (12.1%; 95% CI: 10.46; 13.85), and 57 (3.9%; 95% CI: 2.94; 4.98) would have been diagnosed with prediabetes based on a single HbA1c measurement and without re-measurement as recommended by clinical guidelines. However, the benefit of antihypertensive treatment becomes evident at a BP above 160/100 mmHg (moderate HT) [26] and is less clear in people without CVD and mild HT [27, 28], suggesting that this level should be used as a cut-off value in screening programmes. At this somewhat higher cut-off, 94 women (6.4%) would have been suspected of HT. Based on current evidence, screening for prediabetes is not beneficial in relation to reducing all-cause or cardiovascular mortality although it is associated with a delay in progression to DM [29].

This study informs the debate on the rationale of screening for preclinicial and manifest CVD by substantiating that such screening seems generally accepted judging by the attendance rate. The study also presents an argument for screening for undiagnosed diseases. However, our data do not allow us to recommend a particular preventive screening strategy. Future studies are needed to determine how especially preclinical diseases should be taken into account for designing cost-effective, disease-specific preventive screening strategies. Additionally, the identified hesitation towards prophylactic treatment needs to be investigated further to clarify its causes and to learn how we may address such hesitation from a gender-specific perspective. This observed hesitation raises the question whether prophylactic treatment is considered acceptable by the screened population and, consequently, whether the treatment strategy fulfills the World Health Organization’s (WHO) criteria for acceptable treatment [30]. Furthermore, clarification of the value of adding carotid ultrasound to the CVD risk assessment as well as management of CP as a single risk factor are required to clarify its relevance according to the WHO’s criteria on the importance and cost of case findings.

### Limitations

Although the population-based setup and high attendance rate make selection bias unlikely, our study had some limitations, as does any other observational study. Firstly, we used two-dimensional-based ultrasound, which may be less effective in identifying CP than three-dimensional-based ultrasound, and we may therefore have underestimated the prevalence of plaque as suggested by the fact that Sillesen et al. [31] found CP in 78% of asymptomatic Americans with a mean age of 68.8 years. However, the difference in plaque frequency between their and our study could also be due to the definitions of plaque used. Hence, Sillesen et al. included cIMT ≥1.5 mm in their definition of plaque, whereas we did not classify cIMT ≥1.5 mm as plaque. However, in all women with cIMT ≥1.5 mm, plaque was also identified according to the chosen plaque definitions. Secondly, comorbidities were collected using a self-reported approach, and therefore especially the finding of insufficient OAC should be interpreted with caution as we did not confirm the AF diagnosis with medical records. However, our finding of a low adherence to OAC is in accordance with the literature [32]. Thirdly, screening of AF was based on pulse rhythm when measuring ABPI, which is not validated as a method for AF screening. Fourthly, it is very likely that our study findings cannot be fully extended to other ethnicities and populations with other risk factor profiles such as a higher proportion of obese people, for example. Finally, this study cannot establish whether a screening offer like the one described herein may be recommended as a permanent screening offer. The best evidence for that would be achieved through expensive, large-scaled and long-running randomised trials. Alternatively, the experiences gained herein may be used in models predicting the benefits and cost-effectiveness of the screening offer.

## Conclusion

Screening for CVD and DM is generally acceptable and relevant for elderly women, indicating that a rationale exists for a multifaceted screening programme. However, the identified hesitation towards prophylactic therapy may affect the rationale and the effectiveness of CVD screening, and these critical issues should be addressed in future screening programmes.

## Abbreviations

AAA: Abdominal aortic aneurysm
ABPI: Ankle-brachial pressure index
AF: Atrial fibrillation
AP: Anterior-posterior
ASA: Low-dose aspirin
BP: Blood pressure
CCA: Common internal artery
CI: Confidence interval
cIMT: Carotid intima-media thickness
CP: Carotid plaque
CT: Contrast computer tomography
CVD: Cardiovascular diseases
DM: Diabetes mellitus type 2
ECG: Electrocardiogram
FH: Familial hypercholesterolaemia
GP: General practitioner
HbA1c: Glycated haemoglobin
HT: Hypertension
LDL: Low-density lipoprotein
N: Number
OAC: Oral anticoagulant therapy
PAD: Peripheral arterial disease
SD: Standard deviation
TC: Total cholesterol
WHO: World Health Organization’s
*x*^2^: Chi-squared test
